# How to introduce medical ethics at the bedside - Factors influencing the implementation of an ethical decision-making model

**DOI:** 10.1186/s12910-017-0174-0

**Published:** 2017-02-23

**Authors:** Barbara Meyer-Zehnder, Heidi Albisser Schleger, Sabine Tanner, Valentin Schnurrer, Deborah R. Vogt, Stella Reiter-Theil, Hans Pargger

**Affiliations:** 1 Department of Clinical Ethics, Psychiatric Hospitals and University Hospital Basel, University of Basel, Wilhelm Klein-Strasse 27, 4012 Basel, Switzerland; 2 Department for Anesthesia, Surgical Intensive Care, Prehospital Emergency Medicine and Pain Therapy, University Hospital Basel, University of Basel, Spitalstrasse 21, 4031 Basel, Switzerland; 3 Clinical Trail Unit, Department of Clinical Research, University Hospital Basel, University of Basel, Spitalstrasse 12, 4031 Basel, Switzerland; 4 Institute of Nursing Science, University of Basel, Bernoullistrasse 28, 4056 Basel, Switzerland

**Keywords:** Facilitators, Barriers, Implementation, Medical ethics, Ethical decision-making model

## Abstract

**Background:**

As the implementation of new approaches and procedures of medical ethics is as complex and resource-consuming as in other fields, strategies and activities must be carefully planned to use the available means and funds responsibly. Which facilitators and barriers influence the implementation of a medical ethics decision-making model in daily routine? Up to now, there has been little examination of these factors in this field.

**Methods:**

A medical ethics decision-making model called METAP was introduced on three intensive care units and two geriatric wards. An evaluation study was performed from 7 months after deployment of the project until two and a half years. Quantitative and qualitative methods including a questionnaire, semi-structured face-to-face and group-interviews were used.

**Results:**

Sixty-three participants from different professional groups took part in 33 face-to-face and 9 group interviews, and 122 questionnaires could be analysed. The facilitating factors most frequently mentioned were: acceptance and presence of the model, support given by the medical and nursing management, an existing or developing (explicit) ethics culture, perception of a need for a medical ethics decision-making model, and engaged staff members. Lack of presence and acceptance, insufficient time resources and staff, poor inter-professional collaboration, absence of ethical competence, and not recognizing ethical problems were identified as inhibiting the implementation of the METAP model. However, the results of the questionnaire as well as of explicit inquiry showed that the respondents stated to have had enough time and staff available to use METAP if necessary.

**Conclusions:**

Facilitators and barriers of the implementation of a medical ethics decision-making model are quite similar to that of medical guidelines. The planning for implementing an ethics model or guideline can, therefore, benefit from the extensive literature and experience concerning the implementation of medical guidelines. Lack of time and staff can be overcome when people are convinced that the benefits justify the effort.

## Background

The introduction of new processes and procedures into practice is not easy and presents major challenges for the actors. With awareness that the transfer of knowledge from theory into practice does not happen by itself, important research on modes of implementation in various medical disciplines, e.g. general practice [1], physical therapy [2], and nursing [3] has emerged in the last years. Authors frequently point out the importance of identifying possible facilitators and barriers in advance to adapt the implementation strategy [4–6]. However, few publications give practical advice on how these barriers can first be explored and then be incorporated into the implementation strategy. Gurses et al. have introduced a “Barriers Identification and Mitigation Tool”, which could be helpful [7].

Little research has examined the implementation of ethics guidelines and decision-making models. In 2010, the Swiss Academy of Medical Sciences investigated the distribution and use of some of their medical ethics guidelines by means of a questionnaire distributed among general practitioners and internists [8]. Some papers examine the introduction of clinical ethics support services (CESS). This literature is very relevant as METAP is itself one approach of CESS. In 2001, Slowther et al. reported on the dissemination of CESS in the United Kingdom, especially hospital ethics committees [9]. Obstacles to the successful development and effectiveness of CESS were: lack of resources (financial and human), no availability of training for members, reluctance of clinicians (especially physicians) to recognize and use CESS, and difficulties in raising the profile of the committee within the institution. A survey of all German hospitals investigating the strategies of and experiences with the introduction of CESS found difficulties to introduce the service in half of the hospitals, wherein opposition by some physicians was mentioned most often. There were difficulties with the nomination of committee members and with the development of the terms of reference, while the formation of inter-professional working groups and the adoption of internal processes stood the test [10].

The dissemination of clinical ethics support in hospitals is increasing in somatic as well as in mental health care. A systematic review showed that psychiatry has been, apart from pioneers, more slow to implement CESS on an international scale compared to physical medicine. Existing strengths such as communication skills as well as weaknesses, e.g. a reductionist approach to respond to ethical issues with legal procedures, are discussed as possible reasons as well as lack of resources [11]. Moreover, various clinical fields with different patient groups such as acute or chronic, or the young or old may require specific implementation strategies as well as context-adjusted forms of CESS in order to flourish [12, 13]. Specific tasks and challenges of implementation require specific approaches; ten areas of tackling implementation by rules of thumb are presented by Reiter-Theil distinguishing the difficulties for instalment, respectively [13].

The implementation of new approaches and procedures of medical ethics is as complex and resource-consuming as in other fields, and strategies and activities must be carefully planned to use the available means and funds responsibly. Which facilitators and barriers influence the implementation of *ethics* support and models in daily routine? Are there different promotional and inhibitory factors compared to the introduction of *medical* guidelines? Finding commonalities, the implementation of ethics structures could benefit from the research about medical guideline implementation. Do facilitators and barriers relevantly differ between sites? This paper explores these questions and relates to the introduction of the medical ethical decision-making model METAP in various wards of four Swiss hospitals [14, 15].

## Methods

The presented data are part of a more extensive study that evaluated the acceptance, practicability, and impact of METAP on the structural, product, process, and outcome level.

### The ethics decision-making model METAP

METAP (**M**odular, **E**thical, **T**reatment decisions, **A**llocation of resources at the micro-level, and **P**rocess) is an evidence-oriented ethics decision-making model providing knowledge and procedures for clinical ethics support. A corresponding manual offers descriptions of the ethical principles, rules, and criteria to be followed when facing difficult cases. A short version called Leporello summarizes the core knowledge in a practical form (e.g. checklists for collecting and arranging important information, algorithms for ethical case discussion) [15, 16]. The goal of METAP is to introduce medical ethics into the daily routine. The METAP approach aims at structuring the decision-making process and enhancing the ethical competencies of the clinical staff involved. This is supposed to allow the caregivers to first rely on their own competencies before initiating clinical ethics aconsultation [17].

Rather than relying on a single strategy such as ethics consultation or committee, where available, a four-level escalation model approach has been proposed. Level 1 of the escalating model suggests that a staff member who has an ethical concern will consult the short version and the core knowledge that is given there. At level 2 the staff member calls upon the help of a peer facilitator (trained in ethics) who is a member of the clinical team. For problems of a higher complexity, level 3 foresees an internal interdisciplinary ethical case discussion among the care team on the ward following an explicit procedure described in the manual and supported by tools (e.g. checklists) in the short version. At level 4, if available, a clinical ethics consultation on demand of the team or leadership with (in the ideal case) experienced and qualified ethics consultants is established.

The implementation strategy of METAP includes the provision of training, especially for a small group of care team members nominated to become peer facilitators. These facilitators are familiarized with the manual and trained through modelling and feedback sessions to effectively guide internal ethical case discussions. Other staff members on a ward have become familiar with METAP during information meetings and regularly performed ethical case discussions [16].

### Study design

The evaluation was oriented toward the standards of medical quality management. Quantitative and qualitative methods were used: a questionnaire, semi-structured face-to-face and group-interviews guided by questions derived from the research questions. The purpose of combining quantitative and qualitative methods is defined in the framework of Greene et al. as follows: “In a complementarity mixed-method study, qualitative and quantitative methods are used to measure overlapping but also different facets of a phenomenon, yielding an enriched, elaborated understanding of that phenomenon” [18]. The issues of the questionnaire focused on the structural conditions of the different wards, whereas the interviews allowed collecting the experiences and opinions of the participants. The general approach was checked by an interdisciplinary panel of users and researchers [14]. Table 1 shows the questions analysed in this paper (the whole questionnaire included 51 quantitative and 9 qualitative questions).

**Table 1:** Methods and questions/statements

Method	Questions/Statements	Answer options				
Questionnaire (quantitative)	1. When I am confronted with an ethical problem, there is enough time …					
	a) …to solve the problem with the help of information in the Leporello flyer (level 1).	never	seldom	regularly	very often	NA^a^
	b) …to discuss the problem with a trained peer facilitator (level 2).	never	seldom	regularly	very often	NA^a^
	c) …to solve the problem with an ethical case discussion within the team (level 3).	never	seldom	regularly	very often	NA^a^
	d) …to call and hold an ethical consultation with an external expert (level 4).	never	seldom	regularly	very often	NA^a^
	2. There is enough staff to solve an emerging ethical problem.	no	rather no	rather yes	yes	NA^a^
	3. There is a suitable room in our ward to perform an ethical case discussion.	no	rather no	rather yes	yes	NA^a^
	4. Our medical director supports and actively encourages the use of METAP.	no	rather no	rather yes	yes	NA^a^
	5. Our nursing director supports and actively encourages the use of METAP.	no	rather no	rather yes	yes	NA^a^
Interviews (qualitative)	6. In your opinion, which factors support or hinder the use of METAP? (one-to-one and group interview)					
	7. Do you have the impression that there is enough time to solve ethical problems using METAP? If no, what are the reasons? (group interview only)					
	8. Is there enough staff to solve an ethical problem when it arises? (one-to-one interview only)					

^a^ Not applicable

The local ethics committee judged the project as a quality-assurance measure, and deemed it ethically acceptable.

### Setting and participants

The implementation and evaluation of METAP was performed on three intensive care units and two geriatric wards, with one intensive care unit and one geriatric unit included as a pilot study site between January 2009 and June 2011 (site descriptions see Table 2). During the time period of the study, level 4 (i.e. ethics consultation) was not generally implemented in the involved institutions and was, thus, not readily available. Moreover, ethics consultation service – if practiced – might have been less than professional in some instances. After the study period, ethics consultation was implemented widely upon recommendation of the Swiss Academy of Medical Sciences [19]. The evaluation began in July 2011 and finished at the end of 2011.

**Table 2:** Characteristics of the institutions

Site	Type, disciplines	Number of beds	Time between start of implementation and evaluation
A (pilot ward)	University hospital Surgical intensive care All surgical disciplines (transplantation: kidney only)	22	2 years 6 months
B (pilot ward)	University hospital Acute geriatrics Assessment of geriatric patients with multiple chronic diseases and acute deterioration	28	2 years 6 months
C	Community hospital Rehabilitation and care after cerebrovascular insult Treatment and care for dementia	46	7 months
D	Cantonal hospital Medical and surgical intensive care No cardiac- and neurosurgery, no transplantations Limited therapy of severe respiratory insufficiency	10	7 months
E	Private hospital Medical and surgical intensive care No cardiac- and neurosurgery, no transplantations Limited therapy of severe respiratory insufficiency	6	1 year 5 months

Participants of the face-to-face and group interviews were selected following the “theoretical sampling strategy” [20], taking the individual experience in the use of METAP and participation in at least two ethical case discussions (level 3) as inclusion criteria. The interviewees were selected by asking the nurse manager or the person responsible for the on-site coordination. The characteristics of the participants of the two forms of interviews are listed in Tables 3 and 4. The individual interviews took place before the group interviews.

**Table 3:** Overview participants

Ward	Questionnaire				One-to-one interviews				Group interviews			
	Physicians	Nurses	Others	Total	Physicians	Nurses	Others	Total	Physicians	Nurses	Others	Total
A	9	29	0	35	3	2	0	5	2	3	0	5
B	3	10	0	13	3	2	0	5	2	8	0	10
C	3	11	16	30	3	3	2	8	2	4	4	10
D	1	13	0	14	3	4	0	7	1	7	0	8
E	4	24	2	30	2	5	1	8	2	6	2	10
Total	20	87	18	122	14	16	3	33	9	28	6	43

**Table 4:** Characteristics of the interview participants

Characteristic	Value
Gender	
Female, n (%)	41 (65.1)
Male, n (%)	22 (34.9)
Years of practice	
On the ward, mean (min-max, SD)	8.92 (0.5–39, 9.11)^a^
Total, mean (min-max, SD)	19.48 (1–45, 11.14)^b^
Part-time employment, n (%)	26 (41)^c^
Full-time employment, n (%)	35 (55)^c^
Number of ethical case discussions experienced, mean (min-max, SD)	4.47 (2–20, 3.21)^d^
Number of participants one-to-one and group-interview	
Ward A	3
Ward B	2
Ward C	3
Ward D	2
Ward E	3
Total	13

^a^ Not applicable 2 participants

^b^ Not applicable 1 participant

^c^ Not applicable 2 participants

^d^ Not applicable 5 participants

### Data collection and analytic procedure

The evaluation started with the interviews as soon as the qualitative “saturation criterion after the implementation” was reached, which represents an adequate visibility of METAP on the ward as assessed by the responsible nurse. Visibility means that most of the staff members are knowledgeable about the principles and procedures of METAP and have taken part in at least one ethical case discussion. Use of this criterion was necessary due to the different structural conditions on the wards preventing the option of adhering to the same evaluation period on every ward. Finally, all care team members of all wards received the evaluation questionnaire.

Interviews were conducted face-to-face and were tape-recorded, including detailed notes made by the interviewer. Informed consent was obtained by all interview participants.

#### Qualitative data

The verbatim transcribed interview data were analysed using qualitative content analysis by Mayring [21] and thematic analysis [22]. These two methods allow for identifying and providing a rich, detailed analysis of patterns across a data set. Data were re-read numerous times. Following familiarization, the entire data set was comprehensively coded. Codes were derived from the data in a circular top-down and bottom-up process. In a next step, the codes were combined to form overarching themes, each of which was tested for intra- and interrater reliability to adopt relative system stability by a ratio of ≥0.75. Through this approach, the validity of the data could be consolidated across all wards. Based on work by Leech and Onwuegbuzie [23], and Bryman [24] a pragmatic analysing approach was adopted, which allows to quantify, prioritize, and weight qualitative data. The number of statements per themes and codes was counted and checked for each ward.

#### Quantitative data

In the questionnaire (Table 1), the answer options were “never”, “seldom”, “regularly”, “very often”, “not applicable” for four questions 1 a-d, and “no”, “rather no”, “rather yes”, “yes”, “not applicable” for questions 2–5. Answer options were transformed into a score, ranging from 1 (“never” resp. “no”) to 4 (“very often” resp. “yes”), and the option “not applicable” was coded as “NA”. Mean and standard deviation are presented. Since the questions were formulated as positive statements, a high score indicates that the factor was considered as facilitating the implementation of METAP.

Quantitative data were analysed using the statistical software R (version 3.1.1) [25]

## Results

### Questionnaire

**Fig. 1:**
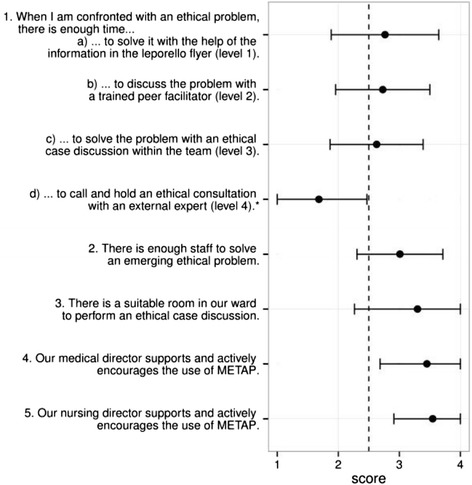
Scores for questions 1–5 (Table 1). Scores for questions 1–5, corresponding to the answers (mean and standard deviation): *Questions 1 a-d:* 1 = never, 2 = seldom, 3 = regularly, 4 = very often; *Questions 2-5:* 1 = no, 2 = rather no, 3 = rather yes, 4 = yes; * Level 4 was not available at all sites

A total of 122 questionnaires were analysed. The response rate was 57%. Overall, participants judged the existing resources considered as necessary for a successful implementation (time, staff, room, and support) to be sufficient (Fig. 1) (over-all average score: 3.0 ± 0.9; mean ± standard deviation).

Concerning time (question 1), the general opinion was that there is enough time to solve an ethical problem with the aid of the Leporello (level 1, question 1a) and discuss it with a member of the facilitating group (level 2, question 1b) or with an internal ethical case discussion (level 3, question 1c). At that time, ethics consultation (level 4) was not at all or not easily available in the hospitals/on the wards included in this study. However, after asked about ethics consultation, most participants wrote that there was never or seldom enough time to call and hold a proper ethics consultation with an invited ethics consultant or committee representative to chair the meeting (level 4, question 1d).

According to the participants, there was sufficient staff on the ward to solve ethical problems (question 2), and a suitable room for ethical case discussions (question 3). Further, medical directors and the nursing management were regarded as actively supporting the use of METAP (questions 4 and 5).

**Fig. 2:**
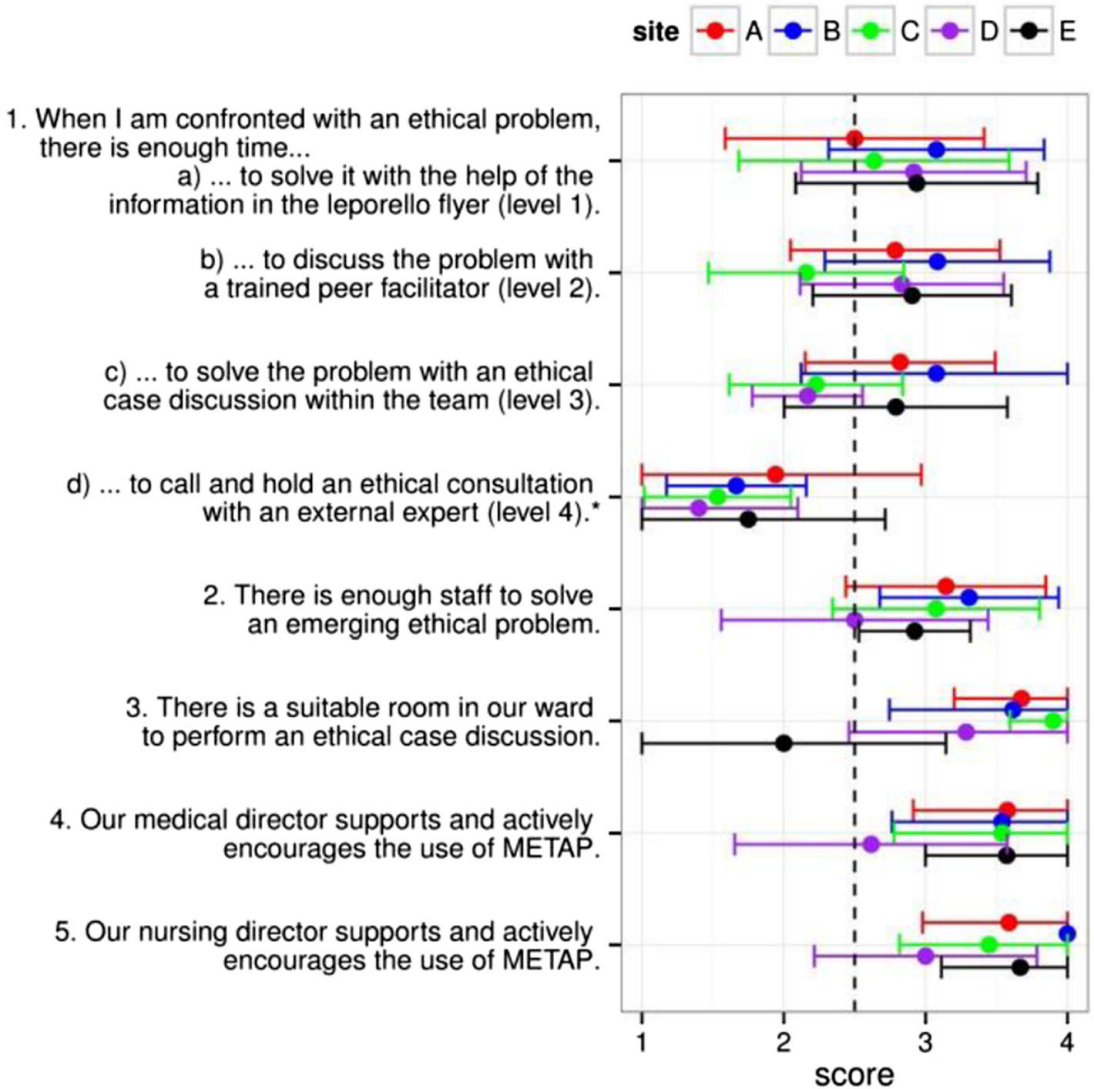
Scores for questions 1–5 (Table 1) divided by implementation sites: Scores for questions 1–5, corresponding to the answers divided by implementation sites (mean and standard deviation): *Questions 1 a-d:* 1 = never, 2 = seldom, 3 = regularly, 4 = very often; *Questions 2-5:* 1 = no, 2 = rather no, 3 = rather yes, 4 = yes; * Level 4 was not available at all sites

The views differed regarding the judgment how strongly each factor was considered as facilitating or hindering the implementation of METAP (Fig. 2). Overall, implementation of METAP was regarded as easiest on wards B (3.2 ± 0.9) and A (3.1 ± 0.9), and most difficult - but still possible - on ward D (2.6 ± 0.9). Ward E was the only one without a room suitable for ethical case discussions (question 3). Support by medical directors and nursing management (questions 4 and 5) was considered weaker on ward D compared to the other four wards.

### Face-to-face and group interviews

A total of 33 face-to-face interviews were conducted with 14 physicians and 16 nurses. In addition, one physical and one activating therapist and a pastor were interviewed. Ten physicians, 28 nurses, and 6 persons of a further profession took part in the 9 group interviews (Tables 3 and 4). A total of 13 persons participated in the face-to-face as well as the group interview. About two-thirds of the participants were female; one-third was male. Mean years of practice on the ward was 8.92 years with a quite large range. More than half of the interviewees were employed full-time.

The answers to the question “In your experience, which factors support or hinder the use of METAP?” could be divided in four main categories, namely “culture/context”, “structures/resources”, “METAP/ethics as such”, and “individual level”. Each of these four groups could be further divided into sub-categories (Tables 5 and 6).

**Table 5:** Factors facilitating or inhibiting the implementation of an ethical decision-making model

	Facilitators, examples	n	Barriers, examples	n
Culture/context				
Clinical situations	Patients are older and sicker. METAP tends to be used if: - Nurses reach their limits - If there is disagreement about the appropriateness of a therapy - If the physician does not know what to do	24	If there are no problems and everything runs smoothly, METAP is used less often. If everyone has the same opinion, METAP is not necessary. High turnover of patients	10
(Ethical) culture, sensibility	Integration in the daily routine Openness for new procedures Existing awareness for ethical questions	17		
Collaboration (interprofessional)	All professional groups need to be motivated and engaged.	5	If a certain profession does not show engagement. Disagreement between the professions	11
Perception of a need for a model	There is a need for legal protection. Waiting for a guideline because of big problems	14		
Support by leadership	Support by providing time and organizing a replacement	10	Lazy and slow hierarchical structures	1
	Total	70	Total	22
Structure/resources				
Time	A discussion in the team is time-saving compared to many one-on-one discussions. It is well invested time.	10	Lack of time (e.g. many very sick patients on the ward) Physicians do not have enough time because a lot of other things have to be done.	34
Staff			Lack of personnel (sometimes an ethical case discussion has to be postponed) Fluctuation of staff due to shift work	15
Fixed dates	Fixed dates are necessary because of shift work.	9	Difficult to find a suitable date for all participants	2
Competition by other projects			Big changes on the ward Many other projects and introductions at the same time	4
	Total	19	Total	55
METAP/ ethics as such				
Presence/awareness	When you know it. Talking about METAP regularly Word of mouth from users	10	If METAP is not known. If it is not used regularly, it is forgotten.	9
Ethics as such	Ethics is a trendy issue. More openness towards ethical themes than in the past	2	Ethics is difficult and abstract. Fear of contact with ethics	8
Acceptance	Acceptance by all hierarchical levels If METAP is noticed to be good and is taken seriously	8	If one thinks “Oh please! Not another new concept!”	2
Effect	It is important to see an effect, a result. If pressure is reduced	6	The impression that it gets nowhere and no change is to be seen	2
Characteristics	Clear and simple presentation in the short version No prescriptions, but a definition of an approach	3	Many prescriptions, which cannot be changed	1
Availability	If the material is available.	2	Material not available on the ward	1
	Total	31	Total	23
Individual level				
Engaged staff members	It takes motivated people, a promoter.	14	The members of the “Steuergruppe” are not known.	1
Individual ethical competence	Existing ethical competence No ethical training	6	No perception of ethical problems Not familiar with ethical questions	8
Attitude personal characteristics	Positive personal attitude	1	Ignorance and lack of motivation No readiness in dealing with a problem	6
	Total	21	Total	15

**Table 6:** Factors facilitating or inhibiting the implementation of an ethical decision-making model divided by implementation sites

	Facilitators						Barriers					
	A	B	C	D	E	Tot.	A	B	C	D	E	Tot.
Culture/context												
Clinical situations	1	10	5	2	6	24		3	2	5		10
(ethical) culture, sensibility	3	5	8		1	17						
Collaboration (interprofessional)				5		5	4			6	1	11
Perception of a need for a model	5	9				14						
Support by leadership	4	1	3		2	10					1	1
Structure/resources												
Time		8		1	1	10	4	5	9	13	3	34
Staff							2	1	3	4	5	15
Fixed dates	3		6			9			2			2
Competition by other projects									3		1	4
METAP/ethics per se												
Presence/awareness		2	2	4	2	10		2	3	4		9
Ethics as such	1	1				2	1	7				8
Acceptance	5	2			1	8		1			1	2
Effect		1		5		6				2		2
Characteristics		1	1		1	3					1	1
Availability			1		1	2			1			1
Individual level												
Engaged staff members	2		3	5	4	14			1			1
Individual ethical competence	1	5				6		3	5			8
Attitude/personal characteristics	1					1	1	1	1	3		6

#### Culture/context

This main category received 92 statements, which is more than one third of all statements. The most common sub-category concerns those clinical situations that make the use of METAP more likely, e.g. numerous very sick patients, uncertain situations, or prevalent disagreement within the team (*n* = 24). In contrast, if everything runs smoothly and there are no problems, METAP seems to be needed less often *(n* = 10). These situations arising from everyday clinical practice determine the need for ethical assistance on an individual or group level, as illustrated in the following quotes:

*I think we use it more often and are more likely to use it whenever there are uncertain situations. (physician, site B)*

*It is facilitating if many in the team do not know what to do and recognize that a case discussion is necessary. (nurse, E)*

An established ethics culture and sensitivity are important facilitating factors (*n* = 17).

*A culture has to develop and this culture, which is beneficial, has developed here. (nurse, B)*

For some wards, it is a problem that not all professional groups show the same engagement (*n* = 11). Disagreements between the professions hinder the use of METAP. Interprofessional collaboration was mentioned as a predominant factor by the members of one ward, in a facilitating as well as a hindering manner.

*It is important that all disciplines cooperate and there are not only the nurses who are dealing with…if it is important to acknowledge that it’s a concern of all. (nurse, D)*

The two pilot wards clearly expressed a general need for having an ethics guideline, which was identified as a facilitating factor (*n* = 14).

*We have waited for such a guideline because there are big problems. (physician, A)*

The interviewees of almost all involved wards emphasized the importance of the support given from the medical and nursing management for implementation (*n* = 10 vs. 1).

*It is surely facilitating if the medical director supports it. I mean it is always this way. If the chief finds something good, the resources are made available. (nurse, A)*

#### Structures/resources

Seventy-four statements were codified in the category “structures/resources”. There was a majority on the side of the inhibiting trait (*n* = 55 vs. 19). The subcategory time received most nominations as an inhibiting characteristic (*n* = 34). Although 10 interviewees (mostly from one ward) mentioned that – after becoming familiar with the model - one can also save time with METAP and that it is time well spent, a perceived lack of time clearly predominates. Moreover it is difficult to immediately schedule an ethical case discussion.

*The organization of a case discussion takes time and energy until you have addressed everything and find a suitable date. This can be a problem. (physiotherapist, C)*

*The time factor is a bad excuse, you can always find time; you come to a decision rather quickly using the instrument. (nurse, B)*

Lack of staff also arose as a common barrier (*n* = 15). No statements indicative of facilitating usage were found in this sub-category. Staff turnover due to the rotation of the physicians and the shift work were mentioned most often.

*The lack of continuity due to short rotations of junior doctors and doctors from other wards being on duty on the ICU and not knowing METAP makes it difficult. (physician, D)*

Fixed dates for ethical case discussions are helpful and necessary due to shift work (*n* = 9).

*…because we established a fixed date, Thursday 2 o’clock. It didn’t work well in the beginning when we didn’t have this date. (nurse, A)*

The interviewees of just one ward mentioned that competition existed with other projects (*n* = 4).

*If there were not so many new projects simultaneously all the time… There is simply too much to do at the moment. (nurse, C)*

#### METAP/ethics as such

This main category consisted of 54 statements, of which 31 had a facilitating and 23 an inhibitory significance. Most statements concerning supportive as well as inhibitory factors were arranged in the subcategory “presence/awareness” (*n* = 10 facilitators vs. 9 barriers). Use is facilitated if one is familiar with METAP and is regularly informed about it. In contrast, it is difficult to make METAP known to a large team. If METAP is not used regularly, its use declines. The following quotes illustrate this category:

*It is beneficial to bring METAP to our attention again and again and to use it regularly in every day practice. (physician, E)*

*People forget about it, its use fizzles out over time. You have to reach somehow, that it is presented over and over again. (activating therapist, C)*

The sub-category “ethics as such” contains more inhibitory factors (*n* = 8 vs. 2). It is noticeable that the interviewees of one ward have special respect for ethics and recognize it as a difficult subject (see Table 6).

*Ethics is a difficult theme. (nurse, B)*

*You have to overcome the fear of contact. (nurse, B)*

The sub-categories “acceptance” and “effect” received almost the same number of nominations (*n* = 8/2 vs. 6/2). If METAP is taken seriously and esteemed as a good tool, its use is promoted. When the users experience an effect, they are more inclined to use it. On the other hand, it is frustrating when no change is apparent.

*It really matters how seriously it is taken by everyone. (nurse, B)*

*It depends on how you judge a new concept; if you find it good or think, oh no, not another new concept. (nurse, E)*

*You must learn the hard way how to reduce pressure by using the procedures. You will ultimately perceive the benefit, and that is a motivation to use METAP the next time too. (physician, B)*

Few interviewees mentioned that the “availability of the material” (*n* = 2 vs. 1) and some “characteristics” of the model (*n* = 3 vs. 1) influence the implementation.

#### Individual level

This main category contains 36 statements. “Engaged persons” was the most frequently mentioned facilitating factor (*n* = 14). A driving force and staff members who like to take part in the ethical case discussion even on a free day are what is needed.

*You need people engaging in it. And these persons should have a certain position within the team to be heard. (physician, C)*

*In part, staff members come in from their leisure just for the case discussions. (nurse, A)*

Lack of ethical competence was also recognized as hindering (*n* = 8). The use of METAP is facilitated if ethical competence is active in a team (*n* = 6).

*I think that if you are working with people who do not know about these themes at all, you will be blocked. These persons are not capable of finding an ethical problem because they cannot identify it. (physician, B)*

Individual characteristics and attitudes such as ignorance and lack of motivation inhibit the application (*n* = 6).

*Not all people want to deal with problems, because it’s easier not to do it. (nurse, D)*

#### Structural factors time and staff (Tables 7 and 8)

**Table 7:** Question group interview

“Do you have the impression that there is enough time to solve ethical problems with METAP? If no, what are the reasons?”^1^				Total
Yes, without reasons			- I think yes. - If we can plan it 1 or 2 days in advance, it is possible.	2
	Level 1 and 2		- For level 2 of course - Level 1 or 2 can be made on the weekend too.	2
Rather no	Level 3	Organizational problems	- Level 3 has to be planned. - All people cannot meet because the team is too big. - First of all, it’s an organisational problem. One could take enough time for sure.	10
		Fast decisions difficult	- Sometimes it is hurried. - Short-term it is difficult. - On a Sunday evening, four of us cannot make 45 min to sit together.	7
		Other priorities	- When resuscitating, you cannot think about METAP - Often other things are more important	5

^1^ Question asked at only two wards (B and D)

**Table 8:** Question one-to-one interview

“Is there enough staff to deal with and solve an ethical problem?”			Total
Yes, without reasons			16
Yes, with reasons/addition	Engaged, experienced people	- There is plenty of experienced and trained staff. - There are many interested and engaged persons.	5
	Organizational	- The nursing leader supports METAP case discussions by replacing staff members.	1
Yes, with restriction	Not always enough staff	- That’s very different. Sometimes yes, sometimes not. - There may still be a little bit more. - Sometimes you have nearly to force it in, but it always has been possible. - If there is not enough staff, you have to shift a case discussion 1 or 2 days.	6
	Depending on the situation	- It is very situational. An ethical case discussion once had to be cancelled due to an emergency. - It may happen that there is not enough time to collect all necessary information in a very unstable patient situation.	3
	Resources are allocated	- It is made possible also with scarce resources.	4
	Difficulties organizing level 3	- Sometimes the fixed dates are difficult because the persons knowing the patient better are not working at that time. - Sometime it is difficult to find a moderator or a suitable date. - It is difficult to prepare the discussion if it takes place on short-notice.	10

Two supplementary questions about time (group interview only, on two wards) and staff (face-to-face interview only) were asked because it was expected from the literature that these two factors would be mentioned very often. No participant of the face-to-face interview answered the question with a clear no, whether there is enough time available to resolve an ethical problem with METAP. Difficulties with time were mentioned especially regarding the activities related to level 3: It can be difficult to organize an ethical case discussion on short-notice and to gather all involved staff members. Often other things have priority.

*So I think, if a problem is acute, and it should be done, I look a little bit at the black side that you can round a group to discuss that. (nurse, D)*

*We have time for it: We could take time for it. (nurse, D)*

No one gave a clear no to the question of whether enough staff are available to resolve an ethical problem using METAP. Almost all respondents answered with yes (15 statements from all wards), some with restrictions. Regarding level 3, organizational problems were mentioned frequently:

*Naturally there is a shortage of time, for example, if you want to do a case discussion. But in fact, it does not fail due to staff problems. (physician, C)*

*… it is difficult to organize an ethical case discussion exactly at the time when the persons who care for the patient are available when the discussion takes place. (physician, D)*

*The problem is that people have other priorities at that moment and only want to sit down shortly to discuss it. (nurse, D)*

In fact, there is not enough staff present and you have to squeeze it into the daily routine. However, it is made possible with limited resources.

*In our judgement it is as important that we make it possible, even with scarce resources. (physician, B)*

## Discussion

The implementation of new approaches into the daily routine of the health care system is complex, and a vast number of determinants influences the process [26]. This article examined the factors that facilitate and inhibit the implementation of a medical ethics decision-making model on different hospital wards. The results of the face-to-face and group interviews with users could be divided into four main categories containing facilitating as well as inhibiting factors: culture/context, structures/resources, the model METAP/ethics as such, and the individual level. A wide range of sub-categories illustrate the mosaic of factors that influence a successful implementation. In the following discussion, we first highlight the facilitating and then inhibiting aspects that need to be kept in mind when planning an implementation strategy – be it a medical ethics decision-making model specifically or clinical ethics support service in general.

### What facilitates the implementation of a medical ethics decision-making model?

The most frequently mentioned facilitating factors were: acceptance and presence of the model, support given by the medical and nursing management, an existing or developing (explicit) ethics culture, perception of a need for a decision-making model, and engaged staff members.

Several research groups developed frameworks concerning the implementation of new procedures and models that can be helpful both for developers of guidelines as well as users. The methodologically and empirically well supported PARIHS (Promoting Action on Research Implementation in Health Services) framework describes a successful implementation as follows: “… successful implementation is a function of the relation between the nature of the evidence, the context in which the proposed change is to be implemented, and the mechanisms by which the change is facilitated” [27]. These three elements “evidence, context, and facilitation” need to be considered simultaneously. They are divided into three to four sub-elements, respectively. The description of each sub-element defines a “positive” (high) or “negative” (low) value or dimension [28, 29]. Some of the elements of the PARIHS framework can be found in our results, and they show these opposite values as well. The element “context” - with the sub-elements culture, leadership, receptive context, and evaluation - corresponds quite well with the factors in our main category structure/resources, and culture/context. In agreement with the “composition” of the PARIHS framework, our results also show that a successful implementation cannot be based on the quality or good evidence of a guideline alone. The environment in which the new approach is to be implemented, including the people working within it, also plays an important role. The different results obtained from the different wards in our investigation substantially illustrate the importance of the environmental and human factors.

The features of a guideline itself affect its application. This obvious fact led to the development of a “guideline implementability framework”, which summarizes the literature in a meta-narrative approach [30, 31]. The framework contains the following domains with sub-elements: usability, adaptability, validity, applicability, communicability, accommodation, implementation, and evaluation. The framework provides a detailed description how a guideline has to be “constructed” for easy use. Our results suggest that the ward’s (ethical) culture and context are most important for implementation success, but the characteristics of METAP itself were also mentioned quite often. Thus, the attractiveness of the model itself is helpful.

Our evaluation data show a substantial benefit regarding the reduction of moral distress among the staff when using METAP on a regular basis [32]. This could motivate and, therefore, facilitate further implementations, when mentioned from the start.

### What inhibits the implementation?

Lack of presence and acceptance, insufficient time resources and staff, poor inter-professional collaboration, lack of ethical competence, and inability to recognize ethical problems were identified as factors inhibiting the implementation of the METAP model. But, the results of the questionnaire showed that the respondents stated to have had enough time and staff available to use METAP. Further, the explicit inquiry concerning time and staff revealed that there is enough time and staff if necessary. Lack of time and staff as an organizational problem due to shift work etc. - can be overcome when people are convinced that the benefits justify the effort. There may, however, be a difference in who considers it necessary or not. Also, this optimistic view may not be generalizable to many institutional environments given the economic differences of national health care systems and types of hospitals.

Many of the studies examining inhibiting factors are of particular interest. Cabana et al. summarized 76 articles investigating factors that hinder physicians from following guidelines; these include lack of: awareness, familiarity, agreement, outcome expectancy, self-efficacy, and motivation; moreover they found: external barriers, guideline-, and environmental factors [33]. Davies et al. examined the perspective of healthcare professionals on clinical engagement in quality improvement [34]. One of the ten research questions addressed facilitators and barriers. A wide range of barriers to the establishment of quality improvement in practice was found, and only a limited list of facilitators (e.g. effective training, modern medical records system, and structured programs). Lack of time and resources are the most commonly cited barriers. Other barriers include problems with group dynamics, lack of a coherent overall plan, and organizational impediments. Cochrane et al. put together 256 articles about barriers occurring in the use of guidelines [6]. They distributed the barriers according to the framework of Cabana et al. [33] and weighted them counting the frequency of occurrence. Lack of knowledge of the guideline, lack of time, and organizational barriers were the most frequently observed barriers. This selection of studies reveals the broad range of barriers for implementation of medical guidelines in the health care sector in general. It also supports some of our results about the implementation of an ethics guideline, a medical ethics decision-making model or CESS. Given the many similarities between the implementation of medical and medical ethics models and guidelines, the planning for implementation of the latter can benefit from the extensive literature concerning implementation of the former.

Some sub-categories, e.g. competition with other projects disturbing the implementation and the individual ethical competence, only emerged on two wards. This observation could be one explanation why implementation interventions are not successful under all circumstances [35]. For the “fine-tuning” and timing of an implementation strategy for an ethics guideline, it is helpful to know whether the staff of a certain ward is open and available for the process, or already engaged in other projects [13]. In that case, it would be better to postpone the implementation of an additional project. Also, an assessment of a ward may reveal that insufficient ethical competence and knowledge are available, in which case, the training of facilitators and other users needs to be adapted.

### Medical ethics decision-making model vs. clinical ethics support

The data show that clinical staff, under certain circumstances, is willing and able to adopt a new medical ethics decision-making model. However, from the staff’s perspectives as covered in our data, the focus must lie more on specific merits or failures of the project to work with the new tools (of the model) rather than on the model or its implementation in general. In many Western countries, hospital ethics committees/clinical ethics support services have focused on developing and strengthening the ethics culture of patient care (see Thematic Issues of MHEP 2008, CQHE 2009, HEC2011, Psychiat Prax 2014, Clin Ethics 2016). Therefore, it is most interesting to acknowledge those aspects of METAP that are different from using hospital ethics committees/clinical ethics consultation as “ethics support”.

Two points are to be highlighted in this regard: 1. the escalating model offering self-help (working under certain conditions such as previous ethics training) with levels 1 to 3 as an alternative to hospital ethics committees/clinical ethics consultation, which had not been established at the hospitals participating in the study at that time; 2. the expectation of study participants that level 4 ethics support, i.e. clinical ethics consultation (by committee or individual), would not be the ideal mode of approach as they considered it to be too slow in availability or too time consuming on their own side.

1): The data show how much the possibility to handle and tackle ethical issues on the ward in a self-reliant manner is appreciated by those involved. Training, guideline tools, and the framework that ethics has a place in daily routine strengthen the professional identity and prevent staff members from feeling helpless vis-à-vis ethical concerns. However, it is well known that groups can develop uncritical self-sufficiency and disregard rational quality criteria, even concerning moral content [36]. Thus, this self-reliance should, according to the METAP concept, be related to quality and competence and ideally benefit from some form of independent feedback and training offered by professionals of clinical ethics. Despite being new, these measures of quality development should be feasible – as the study showed, time and resources can be found when they are considered necessary.

2): Given the lack of personal experience of the respondents with ethics consultation (not available at the time), it is impressive how unanimous their expectations – and prejudice – were formulated. In light of the (by now in one hospital with three study sites) well-established structure and services (hospital ethics committees/clinical ethics consultation) and their swift on-demand availability, the question is raised: what are the advantages or disadvantages of solving ethical problems by means of level 1 to 3 approaches compared to level 4 ethics consultation? While this question has to wait until data will be available before it can be answered, we can hypothesize that the escalating model operates in a context where the benefits of low-threshold ethics approaches (levels 1 to 3) should ideally be complemented by the access to ethics support performed by a professionalized and specialized body functioning independently of the clinical routine (possibly through hospital ethics committees/clinical ethics consultation) as it could be shown that this type of CESS has its merits, also with satisfaction rates of more than 90%. This kind of CESS would also be available to offer support by educational activities or feedback.

### Limitations and strength

Our study has certain limitations. We examined five wards at four hospitals in one country. The results cannot be generalized for all kind of hospitals and other countries. We did not distinguish between the results of the face-to-face and the group interviews. The explorative character of the study seems to allow this combination but has to be kept in mind when interpreting the results. Nevertheless, this is the first study of its type to explore facilitating and inhibiting factors for the implementation of a medical ethics decision-making model. It contains 33 face-to-face and 9 group interviews with 63 participants from different professional groups. This is considerable sample, which allows for careful generalisation.

## Conclusions

Facilitators of and barriers to the implementation of a medical ethics decision-making model occur in the domains culture/context, structures/resources, the model METAP/ethics as such, and at the individual level. They are quite similar to those of medical guidelines. Thus, the planning for implementing a medical ethics model or guideline can benefit from the extensive literature concerning the implementation of medical guidelines. Lack of time and staff can be overcome when people are convinced that the benefits justify the effort.

## Abbreviations

CESS: Clinical ethics support services
CQHE: Journal Cambridge Quarterly of Healthcare Ethics
HEC: Journal HEC-Forum
METAP: Ethical decision-making model **M**odular, **E**thical, **T**reatment decisions, **A**llocation of resources at the micro-level, and **P**rocess
MHEP: Journal Medicine Health Care and Philosophy
PARIHS: Promoting Action on Research Implementation in Health Services
SAMS: Swiss Academy of Medical Sciences
